# Supplementary material for: In Vivo Anatomical Research by 3D CT Reconstruction Determines Minimum Acromiohumeral, Coracohumeral, and Glenohumeral Distances in the Human Shoulder: Evaluation of Age and Sex Association in a Sample of the Chinese Population
Source: J Pers Med. 2022 Nov 1;12(11):1804. doi: 10.3390/jpm12111804 (PMC9694460; doi:10.3390/jpm12111804)
Supplement: Supplementary file 1 [file jpm-12-01804-s001.zip › jpm-1932019-supplementary.pdf]

Table S1. Statistical analysis of AHD, CHD and GHD during internal and external rotation of humerus in males and females.

Sex	AHD		CHD		GHD	
	F	*P* value	F	*P* value	F	*P* value
Male	0.024	0.681	0.136	0.914	0.268	0.678
Female	4.090	0.079	0.632	0.916	0.175	0.257

AHD acromiohumeral distance, CHD coracohumeral distance, GHD glenohumeral distance.
